# Diagnosis announcement among mothers of children with leukemia

**DOI:** 10.1192/j.eurpsy.2023.2005

**Published:** 2023-07-19

**Authors:** S. Dhakouani, M. Karoui, N. Hamrouni, R. kammoun, F. Ellouze

**Affiliations:** psychiatry, Razi hospital, tunis, Tunisia

## Abstract

**Introduction:**

The diagnosis of leukemia in a child is a difficult moment for the therapist and for the parents. Nevertheless, this moment is crucial and determining in the course of care and the therapeutic relationship.

**Objectives:**

Determine the quality of diagnosis announcement among mothers of children with leukemia.

**Methods:**

A cross-sectional study was conducted at Aziza Othmana hospital department of haematology in Tunisia between June and July 2021.

We have questioned the mothers about the announcement of the diagnosis: the space frame, the time provided and the availability of the doctor.

**Results:**

We included 31 mothers, their middle age was 41 years old. Acute lymphoblastic leukemia is the most frequent type of cancer in our sample (94%).

According to 4 mothers (13.3%), the diagnosis of leukemia was not announced before the start of treatment.

The quality of the diagnostic announcement was judged to be good in 40% of cases (n=12), average in 12.7% (n=8) and mediocre in 20% of mothers (n=6).

The space frame of the announcement was perceived as appropriate with respecting confidentiality in 18 mothers (69.2%).

The time provided for the announcement was considered sufficient for 17 mothers (65.4%).

The doctor who announced the diagnosis was described as available by 69.2% of the mothers and unavailable by 30.8% of the mothers.

**Conclusions:**

The quality of the diagnosis announcement amoung mothers of children with leukemia in our context is not optimal. Oncologists must be trained in diagnostic announcement and must be aware of the importance of this moment in the subsequent therapeutic relationship.

**Disclosure of Interest:**

None Declared

